# Carbon Fiber Ultramicrodic Electrode Electrodeposited with Over-Oxidized Polypyrrole for Amperometric Detection of Vesicular Exocytosis from Pheochromocytoma Cell

**DOI:** 10.3390/s150100868

**Published:** 2015-01-06

**Authors:** Li Wang, Huiren Xu, Yilin Song, Jinping Luo, Shengwei Xu, Song Zhang, Juntao Liu, Xinxia Cai

**Affiliations:** 1 State Key Laboratory of Transducer Technology, Institute of Electronics Chinese Academy of Sciences, Beijing 100190, China; E-Mails: wangli2011iecas@163.com (L.W.); xuhuiren_2012@163.com (H.X.); ylsong@mail.ie.ac.cn (Y.S.); jpluo@mail.ie.ac.cn (J.L.); swxu@mail.ie.ac.cn (S.X.); zhangsong0112@126.com (S.Z.); liujuntao@mail.ie.ac.cn (J.L.); 2 University of Chinese Academy of Sciences, Beijing 100190, China

**Keywords:** amperometry, ultramicrodic carbon fiber electrode, over-oxidized polypyrrolenanoparicle, vesicular exocytosis

## Abstract

Vesicular exocytosis is ubiquitous, but it is difficult to detect within the cells' communication mechanism. For this purpose, a 2 μm ultramicrodic carbon fiber electrode was fabricated in this work based on electrodeposition with over-oxidized polypyrrole nanoparticle (PPyox-CFE), which was applied successfully for real-time monitoring of quantal exocytosis from individual pheochromocytoma (PC12) cells. PPyox-CFE was evaluated by dopamine (DA) solutions through cyclic voltammetry and amperometry electrochemical methods, and results revealed that PPyox-CFE improved the detection limit of DA. In particular, the sensitivity of DA was improved to 24.55 μA·μM^−1^·μm^−2^ using the PPyox-CFE. The ultramicrodic electrode combined with the patch-clamp system was used to detect vesicular exocytosis of DA from individual PC12 cells with 60 mM K^+^ stimulation. A total of 287 spikes released from 7 PC12 cells were statistically analyzed. The current amplitude (I_max_) and the released charge (Q) of the amperometric spikes from the DA release by a stimulated PC12 cell is 45.1 ± 12.5 pA and 0.18 ± 0.04 pC, respectively. Furthermore, on average ∼562,000 molecules were released in each vesicular exocytosis. PPyox-CFE, with its capability of detecting vesicular exocytosis, has potential application in neuron communication research.

## 1. Introduction

Vesicular exocytosis, the process whereby transmitter-laden intracellular vesicles fuse with the cell membrane and release their contents to the outside of the cell, has been recognized as critical to understanding the mechanism of cell-to-cell communication [1,2]. In practice, however, real-time monitoring the time-temporal response conveyed by vesicular exocytosis is difficult, probably due to its fast reaction, which is usually completed within milli-/micro-seconds [3–6].

A number of technologies have been used for vesicular exocytosis detection with different kinds of microelectrodes to achieve these ideal properties, such as by utilizing micro/nano microelectrodes, micro/nanofabrication, novel detection methods, or microfluidic process [7–12]. Carbon fiber microelectrodes (CFE) with a cone-shaped tip of about 5 to10 μm in diameter, first introduced by Wightman, have now extensively been used to electro-chemically detect exocytosis of transmitters, such as catecholamines [2,10]. The CFE is placed immediately adjacent to a cell so that each packet of transmitter that is released from the cell produces a spike of amperometric current when the transmitter is oxidized on the electrode surface [13–20]. Chen *et al.* developed a microchip with gold electrode modified micro wells. With this chip, it was possible to detect the majority of secreted catecholamines from single adrenal chromaffin cells [21]. Spégel *et al.*, a mercaptopropionic acid modified interdigitated electrode structure was used for low noise recordings of dopamine exocytosis without any advanced positioning schemes [22]. Gillis's group made an automated targeting of cells to electrochemical electrodes for quantal exocytosis [3,23]. Researchers are often faced that how to reduce the noise interference, which easily floods the oxidation current of dopamine released from a single cell. Modifying the surface of microelectrode would be one of the efficient ways to increase its electroanalytical activity.

Polypyrrole (PPy) is a kind of conducting polymer [24,25], as it exhibits high electrical conductivity (ranging from 2 to 100 S/cm), good environmental stability, and biocompatibility. PPy with different nanostructures, such as nanowire, nanotube, and nanoparticle, has been utilized as a matrix to embed or disperse various metal nanoparticles for use in sensors and electrocatalysts [26–30].

By taking into account the abovementioned issue, we here introduce a CFE electrodeposited with over-oxidized polypyrrole nanocomposites (PPyox-CFE) for detecting the vesicular exocytosis from a single cell.

## 2. Experimental Section

### 2.1. Apparatus and Reagents

PC12 cells were cultured in a CO_2_ incubator (SANYO, Osaka, Japan). Original PC12 cells were purchased from Peking Union Medical College Hospital (Cell resource center, CAMS/PUMC, China). PPyox-CFE, a working electrode, was made from pullers (P-97, Novato, CA, USA). Cyclic voltammetry and chronoamperometry were performed on an Autolab PGSTAT302N electrochemical workstation (Autolab, Herisau, Switzerland). The signals of exocytosis detected by CFE were processed on a Patch clamp system (HEKA, Lambrecht, Germany). The carbon fiber was fabricated by Horizontal electrode puller (Sutter Instrument, Novato, CA, USA). The analysis software was Igor Pro 6.1 (WaveMetrics, Lake Oswego, OR, USA). All reagents were used as received without further purification. Water was purified through a Michem ultrapure water apparatus (Michem, Chengdu, China, resistivity >18 MΩ). Pyrrole (Py) was purchased from Sigma-Aldrich Co., Ltd. The phosphate buffer saline (PBS, 0.1 M Na_2_HPO_4_-NaH_2_PO_4_-KCl, pH 7.4) was prepared from a PBS tablet (Sigma). For *in vitro* calibrations, Dopamine solutions were prepared in a phosphate buffered saline (PBS) solution, 0.1 M, pH 7.4 just before use because of their gradual decomposition. For PC12 quantal exocytosis detection, the standard cell buffer solution consisted of (in mM): 150 NaCl, 2 CaCl_2_, 1.2 MgCl_2_, 5 KCl, 11 glucose and 10 HEPES, pH 7.2, freshly prepared prior to use. The high K^+^ solution consisted of (in mM): 150 NaCl, 2 CaCl_2_, 1.2 MgCl_2_, 100 KCl, 11 glucose and 10 HEPES, pH 7.2, freshly prepared prior to use.

### 2.2. Fabrication of CFE

Carbon fiber electrode with diameter of 22 μm was welded together to copper wire with length of 10 cm. Afterwards, the copper wire and carbon fiber were inserted into a glass capillary and pulled in a horizontal electrode puller under the special condition (Heat 525 Pull 70 Vel 70 Time 50). Most of the exposed CFE was sealed within the molten glass micropipette while a blue flame of alcohol burner etches the tip of the electrodes.

### 2.3. Preparation of CFE Modified PPyox

6.94 μL Pywas sonicated in 20 mL DI water for 15 min, and then 25 mg Poly sodium-p-styrenesulfonate (PSS) (Mw = 70,000) was added into the mixture with ultrasonication for 10 min. In the above steps, nitrogen was blown continuously into the solution. The CFE was polished by acetone, ethanol, and deionized (DI) water. Electrochemical deposition of PPyoxon twenty CFEs was performed using cyclic voltammetry in a potential range of −0.1 to 0.85 V *versus* Ag|AgCl at a scan rate of 50 mV/s in a period from 1 to 20 cycles, respectively. The obtained microelectrodes were gently washed with DI water to remove excess non-adsorbed species.

### 2.4. Electrochemical Measurements

The PPyox-CFE performance was carried out with an Autolab workstation connected to a computer. A three-electrode system with the PPyox-CFE as the working electrode, Ag|AgCl wire as reference electrode and a platinum wire as the counter electrode was used. Cyclic voltammetry scanning and chronoamperometry were applied to the three-electrode system. Once the background current reached a steady-state value, different doses of prepared DA was injected into the in PBS under magnetic containment with vigorous stirring and response currents were recorded with time at a constant applied potential. All experiments were conducted at room temperature.

### 2.5. Cell Preparation

PC12 Cells were kindly provided by Peking Union Medical College Hospital (Cell resource center, CAMS/PUMC, China). 2 mL PC12 cells at a density of about 10^6^ cells per mL were originally cultivated into T25 polystyrene cell culture flasks in 6 mL of culture media (Ham's F12K medium supplemented with 10% fetal bovine serum and 5% Horse serum) at 37 °C in a humidified atmosphere of 5% CO_2_/95% air. PC12 cells were cultivated for 24 h before detection of quantal exocytosis experiment.

### 2.6. Measurement and Analysis of Quantal Exocytosis

An EPC-10 patch-clamp amplifier with Patchmaster software was applied for recordings at room temperature. Amperometric measurements were performed at a sampling rate of 10 kHz and the potential of working site was maintained at 650 mV *vs.* Ag|AgCl. Data analysis of quantal release was performed running on IgorProv. 6.1 (Wavematrics, LakeOswego, OR, USA) using threshold method. A quantal exocytosis event was defined as an amperometric spike being six times the standard deviation of the noise background.

## 3. Results and Discussion

### 3.1. Characterization of PPyox-CFE

**Figure 1.**
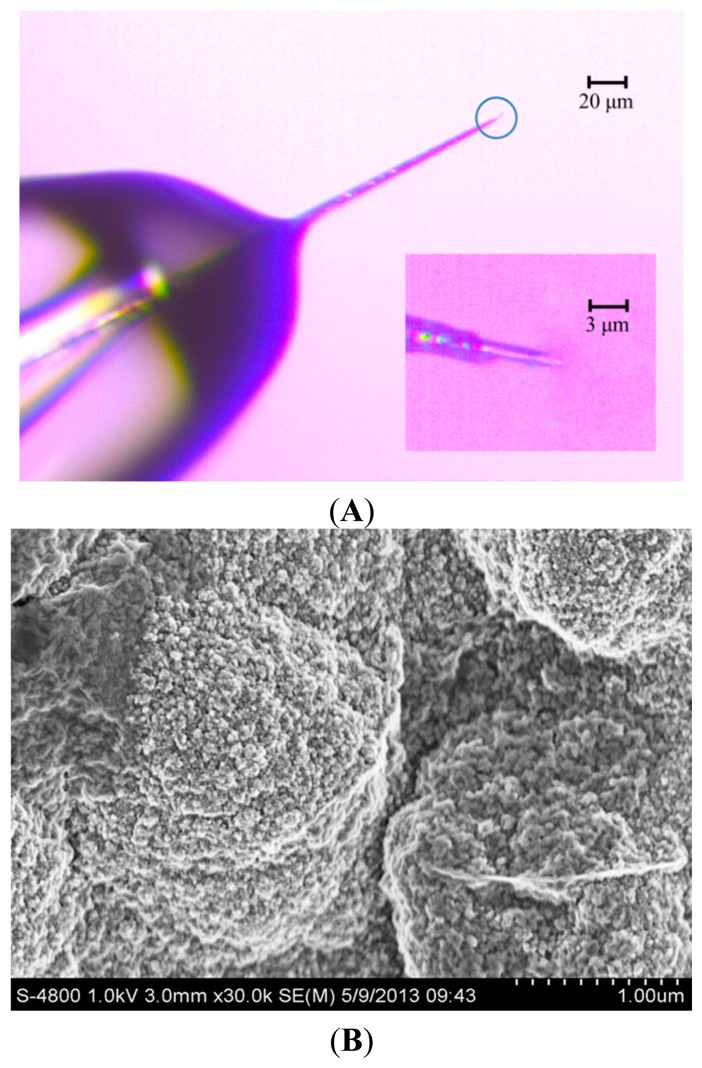
(**A**) The fabricated CFE with diameter of 2 μm; the inset shows interface between carbon fiber and the glass insulator; (**B**) The SEM at the cycle of 8.

The detection limit of vesicular exocytosis from PC12 cell is influenced deeply by the background noise, which depends on electroactive surface area of the working electrode [2,21]. To reduce the background noise, the conductive portion of microelectrode tip should be as short as possible so that the tip can be positioned close to PC12 cell. Figure 1A shows the CFE, whose conductive length was 5 μm and the diameter was 2 μm. The inset in Figure 1A shows the interface between carbon fiber and the glass insulator. The rest of the exposed CFE was well sealed with molten glass micropipette while the tip of the electrode was cone shaped by blue flame etching from an alcohol burner.

Figure 1B shows the SEM of PPyox nanoparticles at cycles of 8 on the tip of CFE. After the modification of the surface with PPyox film, rough surface morphology was obtained with irregular pellets. PPyox nanoparticles were grown densely which were in a spherical shape with a nonuniform diameter of 30–50 nm. Moreover, A number of PPyox nanoparticles aggregate to form larger particles. This implies that the membrane on the electrode may exhibit some nano-effects, such as catalysis.

**Figure 2.**
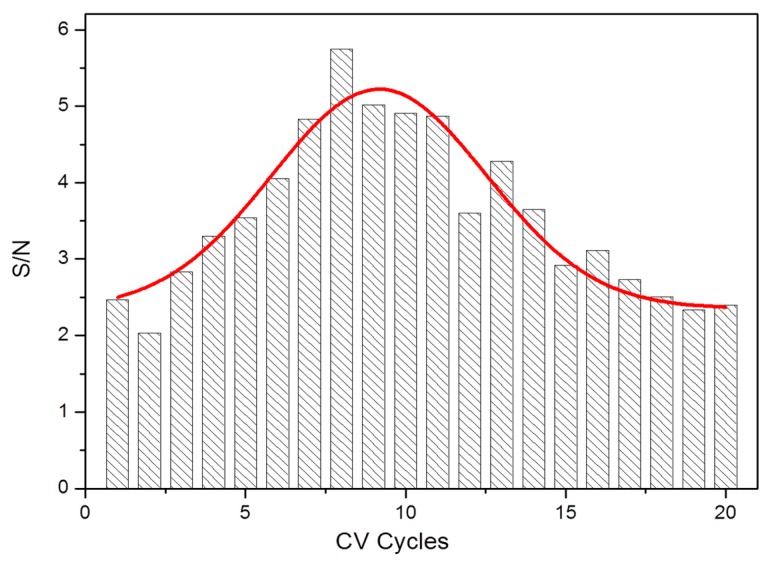
Signal-to-noise (S/N) statistics of current response to 2 μM DA.

In order to properly improve dopamine sensitivity and detection limit with CFE, PPyox nano-film have been attempted to modify the microelectrode. Twenty CFEs were electrodeposited with PPyox using cyclic voltammetry in a period from 1 to 20 cycles, respectively. We identified the optimal electrodeposition condition through signal-to-noise (S/N) statistics of their current response to 2 μM DA solution, as shown in Figure 2. S/N, an important parameter of measuring the anti-noise ability, is the ratio of response current and baseline. The increment of S/N grew with more electrode position cycles up to 8. At cycles of 9 or more, the S/N decreased. Furthermore, the population of S/N is an approximate Gaussian distribution. These results may be attributed to the increase in membrane thickness of PPyox as the number of electro-deposition cycle rises, so that more produced nanoparticles and holes could bring in more noise while the sensitivity improves. Thus, we determined that 8 cycles of electrode position is optimal to achieve the best sensitivity and detection limit of DA.

### 3.2. Cyclic Voltammetry Measurements of DA Using PPyox-CFE

**Figure 3.**
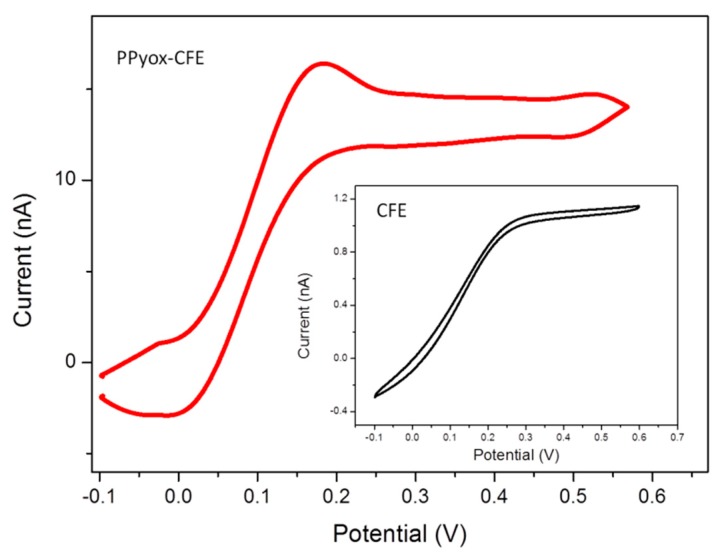
Cyclic voltammetry response of bare CFE was compared to those CFE modified with PPyox the electrochemical in 2 μM DA solution.

Cyclic voltammetry response of bare CFE was compared to those CFE modified with PPyox in 2 μM DA solution, as shown in Figure 3. In addition, the peak currents increased 10.3 times compared with bare CFE, a well behaved reversible redox wave of DA were also obtained from the CFE electrodeposited with PPyox film. The reduction and oxidation peak potential of DA was at 21.3 mV and 149 mV, respectively. Furthermore, the PPyox-CFE also exhibited a highly reproducible response, the reduction and oxidation peak were nearly identical when the modified CFE was used for 6 cycles, suggesting that the surface of CFE with PPyox nanoparticles most likely has a capability of maintaining stable dopamine concentration [13]. Additionally, as a kind of nanoparticle, PPyox, which could achieve a lower oxidation potential (149 mV) of DA, exhibited excellent catalyzing ability towards it [31]. Taking a look at the morphology, the materials and the structure of the PPyox film may help to explain the unexpected higher electro-activity of PPyox-CFE. The polymerization process generated a relatively homogeneous nanostructure size distribution and a rather rough surface, which improve the catalytic activity of the electrode and help to improve the current response and reduce the oxidation peak potential of the electroactive neurotransmitters resulting in the separation of oxidation peak [32–36].

### 3.3. Amperometric Responses to DA

**Figure 4.**
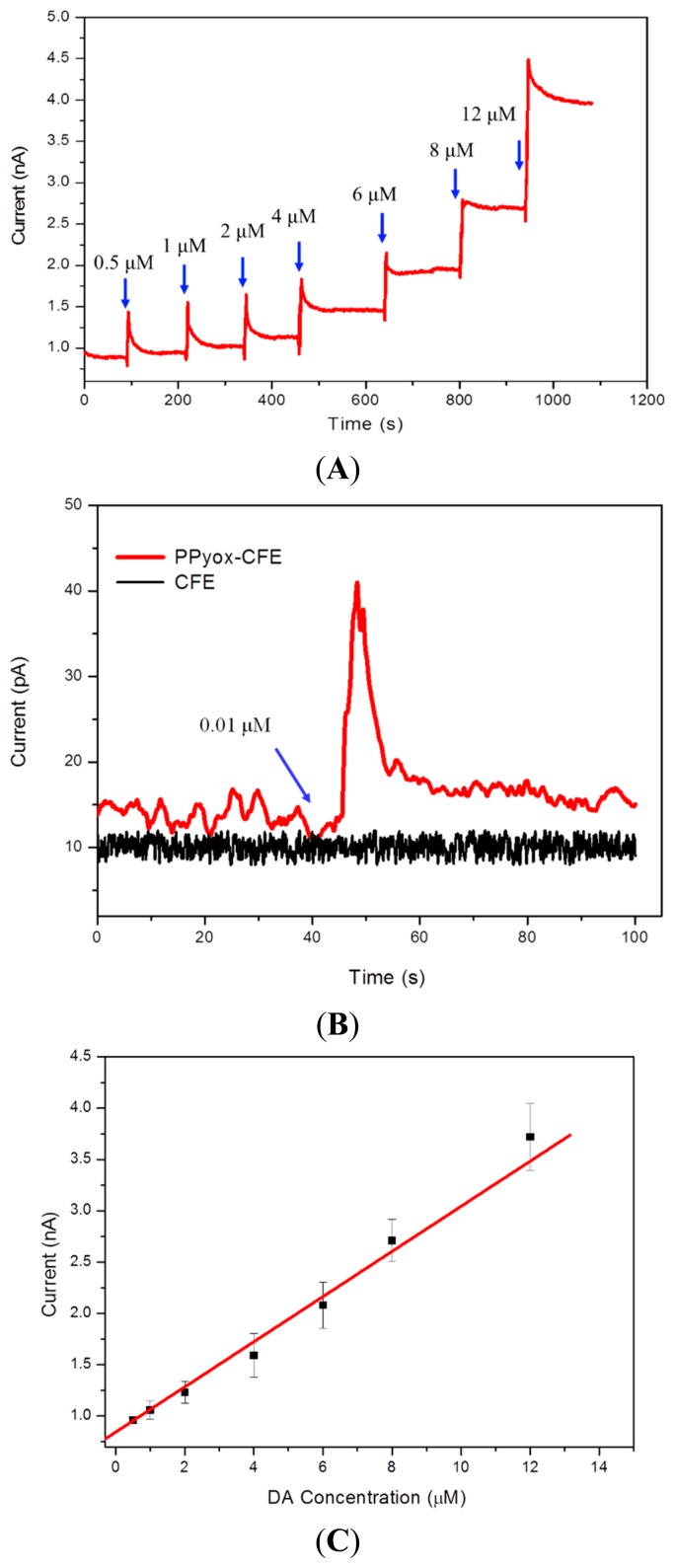
(**A**) Current-time response of the CFE electrodeposited with PPyox film at different concentrations at 149 mV *vs.* Ag|AgCl; (**B**) Detection limit of DA using PPyox-CFE; (**C**) Plot of current *versus* concentration of DA in the range of 0.5 μM to 12 μM.

In order to determine the sensitivity using the CFE electrodeposited with PPyox film, Stepwise addition of aliquots of DA to a stirred solution resulted in stepwise increase of anodic current as described in Figure 4A. Chronoamperometry was employed to detect DA at the potential of 149 mV obtained by the cyclic voltammetry measurement. As shown in Figure 4C, the oxidation current of the DA increases linearly with concentrations in the range of 0.5 μM to 12 μM, with a correlation coefficient of 0.994. Since the surface area of the microelectrode is 22.65 μm^2^, the detection sensitivity of the PPyox-CFE is 24.55 μA·μM^−1^·μm^−2^. To study the detection limit of DA with the CFE, we compare I-T curves of the CFE with that of PPyox-CFE. Figure 4B shows the DA detection limit of 0.01 μM (S/N = 3.3) using PPyox-CFE, however, there is no obvious change in the process of adding DA solution as using CFE. The results demonstrated a low detection limit of the prepared PPyox-CFE, indicating the advantage of the polymerization process generated a rather rough surface, which dramatically elevated the amperometric response of PPyox-CFE by enlarging the effective area.

### 3.4. Recording of Vesicular Exocytosis

PC12 cells, which can synthesize neurotransimitters (mainly DA), garner catecholamine-containing vesicles, set free and pass to receptors of postsynaptic membrane, have been generally used as a model for research [13,25]. It is familiar to us that an increased extracellular high concentration of K^+^ provokes the depolarization of dopaminergic neurons and so triggers exocytosis by opening specific-binding Na^+^ entry, which induces the subsequent opening of voltage-sensitive Ca^2+^ channels. The opening of Ca^2+^ channels allows a quick influx of Ca^2+^ concentration in the intracellular to a level sufficient to provoke the transferring of neurotransmitter-containing vesicles to the plasma membrane for exocytosis [2,10–13,17–21].

**Figure 5.**
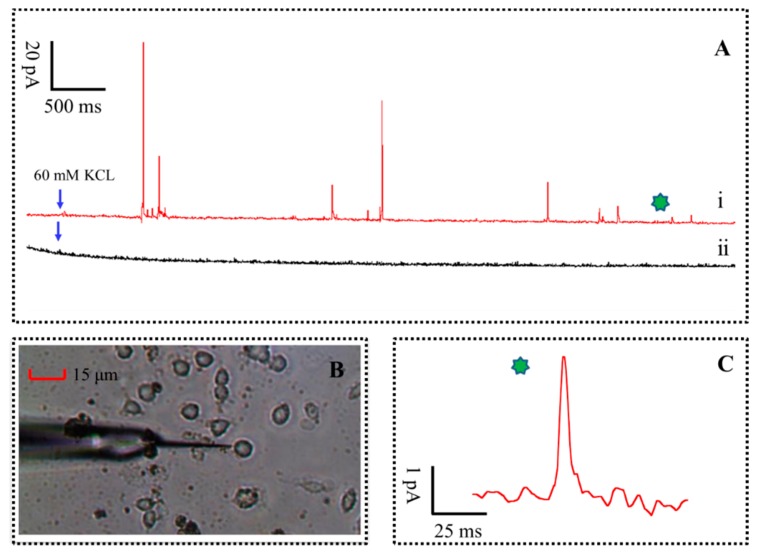
(**A**) The traces are recorded by amperometric method continuously for 25 s at the distance of 1 μm (i), 500 μm (ii) from cell with 60 mM K^+^ stimulation solution, respectively; (**B**) The diagram of the single PC12 cell release monitoring with the PPyox-CFE; (**C**) The obtained minimum amplitude of the spike.

In order to ascertain whether vesicular exocytosis was caused by high concentration of K^+^ stimulation solution (that consists of 150 mM NaCl, 2 mM CaCl_2_, 1.2 mM MgCl_2_, 60 mM KCl, 11 mM glucose and 10 mM HEPES, pH 7.2), two group tests were performed at a sampling rate of 10 kHz, as shown in Figure 5A. Briefly, the experimental results are recorded by amperometric method continuously for 25 s at the distance of 1 μm, 500 μm from cell with 60 mM K^+^ stimulation solution, respectively. The diagram of the single PC12 cell release monitoring with the PPyox-CFE is shown in Figure 5B. The tapered and small size of the PPyox-CFE was planned for direct recordings of a single PC12 cell in actual time. A typical amperometric trace of the test result, red line in Figure 5A, was recorded. As described, a succession of rapid ascended and slow decay of spikes were detected after the injection of high K^+^ stimulus to trigger exocytosis. The minimum amplitude of the spike was 4.9 pA with the S/N of 10.2. However, no obvious spike was recorded with the CFE at the distance of 500 μm from cell.

The parameters of pikes were calculated and analyzed according to the method [26,27]. T (pulse width for the current response of quantal exocytosis) represent the kinetics of the major DA content released from the membrane to the microelectrode surface, whereas I_max_ (the amplitude of the spike) and Q (The charge generated when neurotransmitter release each time) represent a measure of the vesicular DA content, is calculated according to the equation: $Q=\int_{0}^{T}I\;dt$ , all parameters were presented as mean ± SEM (standard error of the mean), respectively. Two hundred eighty-seven Spikes released from seven PC12 cells were statistically analyzed. The mean T, I_max_ and Q of the amperometric spikes from the DA release in a PC12 cell stimulated is 19.6 ± 2.5 ms, 45.1 ± 12.5 pA and 0.18 ± 0.04 pC, respectively. For the release of catecholamine, the number of transmitter molecules, N, is calculated as N = Q/(n × Q_e_) = Q × 3.121 × 10^6^ (molecules/pC), where Q_e_ is the charge of a single electron (1.602 × 10^−19^ C) and n is the number of electrons donated by each oxidized molecule, which equals two for DA. The number of transmitter molecules N can be calculated as ∼562,000 molecules.

### 3.5. Stability of CFE Modified with PPyox Film

**Figure 6.**
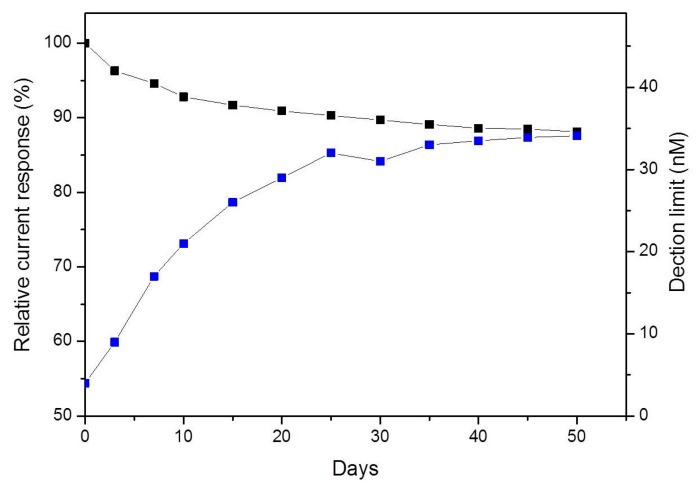
Stability of PPyox-CFE, expressed as percentage of sensitivity to DA in PBS (pH 7.4) and the detection limit of DA.

The stability of the PPyox-CFE was studied by keeping the CFE in PBS at 4 °C for four weeks after having been used for detecting DA release from single PC12 cell. Over the first seven days, the sensitivity of DA decreased by 5.4% and the detection limit was 17 nM. The sensitivity of DA maintained 88.1% of the original sensitivity value after 50 days as shown in Figure 6. These results indicate that the CFE electrodeposited with PPyox film displayed stable and excellent response to DA.

## 4. Conclusions

In this paper, we fabricated a novel carbon fiber microelectrodes based biosensor for real-time monitoring the DA release *in vitro*. Over-oxidized polypyrrole was modified on the working electrodes using electrodeposition, which significantly increased active surface area for DA absorption. Due to this, PPyox-CFE exhibited high sensitivity and low detection limit of dopamine as indicated by the detection of nanomolar dopamine concentrations. Using the PPyox-CFE, we succeeded in recording real-time, high S/N measurements of DA release from cultured single PC12 cell. These results suggest that our PPyox-CFE have broad potential for applications in both basic neuroscience research and the potential for studying the mechanisms of vesicular exocytosis.
